# Pilot Study for Assessing Nontechnical Skills in Emergency Medicine Residents: Why We Should C.A.R.E.

**DOI:** 10.31486/toj.21.0086

**Published:** 2022

**Authors:** Terrell S. Caffery, Claude D’Antonio, Debbra Pogue, Mandi W. Musso

**Affiliations:** ^1^ Emergency Medicine Residency Program, Louisiana State University Health Sciences Center, School of Medicine, Baton Rouge Campus, Baton Rouge, LA; ^2^ Division of Academic Affairs, Our Lady of the Lake Regional Medical Center, Baton Rouge, LA

**Keywords:** *Communication*, *emergency medicine*, *internship and residency*, *physician-patient relations*

## Abstract

**Background:** The growing regulatory and hospital focus on patient experience and patient satisfaction is evidenced by the Centers for Medicare and Medicaid Services implementation of Hospital Value-Based Purchasing and by the Accreditation Council for Graduate Medical Education milestones. However, there is a paucity of data examining the education and evaluation of emergency medicine residents’ nontechnical skills (eg, communication and situational awareness) as they relate to patient interactions. The purpose of the current study was to evaluate a nontechnical skills rating tool with emergency medicine residents during their interactions with patients.

**Methods:** As part of the educational initiative, the authors consulted with a hospitality training and measurement company, the Freeman Group, that developed and trained faculty on the use of an observational tool to assess physicians’ nontechnical skills. Nontechnical skills were assessed in 4 domains designated by the acronym C.A.R.E.: *connect* with the patient, *adjust* the interaction to meet patient needs, *resolve* patient requests, and *empathize* with the patient. Faculty observed emergency medicine residents as they interacted clinically with patients in the emergency department and rated them on a binary scale: acceptable or unacceptable.

**Results:** Thirty-four of 36 residents were observed. Our study demonstrates that the residents performed very well on domains of empathy, adjusting to patients’ knowledge, and resolving requests. However, residents’ abilities to customize conversations to patients (eg, addressing patients appropriately and establishing and maintaining rapport) were rated as unacceptable 31% of the time.

**Conclusion:** Overall, residents performed well on most aspects of nontechnical skills observed during their interactions with patients. However, even when residents were mindful of faculty observing nontechnical skills, they performed unacceptably in their communication with patients in approximately one-third of the interactions. This study provides important insight into nontechnical skill areas that may be influenced with intervention to improve patient interactions, and ultimately, influence patient satisfaction.

## INTRODUCTION

Nontechnical skills in medicine have been defined as “the cognitive, social, and personal resource skills that complement technical skills, and contribute to safe and efficient task performance.^1^” Nontechnical skills include communication, teamwork, situational awareness, decision-making, task allocation, and management of stress and fatigue.^2^ The health care industry has worked to adapt crisis resource management techniques from the airline industry to improve teamwork and avoid potentially hazardous errors.^3^ Authors of a systematic review of articles that examined nontechnical skills training developed the following content themes: communication, error, systems, teamworking and leadership, and situational awareness.^4^ Tools developed to assess nontechnical skills relevant to emergency medicine include the Team Emergency Assessment Measure (TEAM)^5^ that examines domains of leadership and teamwork in emergency response team members and the Observational Skill-based Clinical Assessment tool for Resuscitation (OSCAR)^6^ that examines domains of communication, co-operation, co-ordination, leadership, monitoring, and decision-making during resuscitations. Much less research has focused on emergency medicine physicians’ nontechnical skills during interactions with patients.

Locke et al showed that patients’ perceptions of emergency department (ED) physicians’ communication and interpersonal skills were associated with patient satisfaction independent of hospital length of stay or severity of illness.^7^ In a study of patients’ perspectives of ED nurse and physician interactions with patients, 2 themes emerged: foundational (eg, body language and courtesy) and relational (eg, reassurance, humanism, attentiveness, and politeness) aspects of interaction.^8^ A 2018 literature review identified communication, wait times, and staff empathy and compassion as the most common drivers of patient satisfaction.^9^

Highlighting the importance of nontechnical skills and patient communication is the Centers for Medicare and Medicaid Services (CMS) implementation of Hospital Value-Based Purchasing. Medicare Severity Diagnosis-Related Group payments are withheld and redistributed based on hospital performance on core measures. Thirty percent of the overall score is based on patient satisfaction.^10^ Hospital Value-Based Purchasing has shifted some of the focus in health care to consumer-driven models. In addition, the Accreditation Council for Graduate Medical Education (ACGME) milestones involve nontechnical skills, including practice-based performance improvement, patient-centered communication, team management, professional values, accountability, patient safety, and systems-based practice.^11,12^

As evidenced by the CMS and ACGME initiatives, regulatory and hospital focus on patient experience and patient satisfaction is growing. However, a paucity of data examines the education and evaluation of emergency medicine residents’ nontechnical skills as they relate to patient interactions. The purpose of the current study was to evaluate the reliability and validity of a nontechnical skills rating tool with emergency medicine residents during their interactions with patients.

## METHODS

### Study Population

This pilot study was an educational initiative implemented by an emergency medicine residency program affiliated with a large academic medical center in the southern United States. Emergency medicine residents were observed during patient interactions to measure their nontechnical skills. Residents provided informed consent for the use of their data. This study was approved by the Louisiana State University Health Sciences Center Institutional Review Board.

### Observational Tool Development

The authors consulted with a hospitality training and measurement company, the Freeman Group,^13^ to develop a proprietary observational tool using the internet-based platform Qualtrics^14^ to assess physicians’ nontechnical skills during their interactions with patients. Faculty and members of the Freeman Group met to discuss the emergency medicine milestones and the need for tools to assess residents’ nontechnical skills. Faculty and Freeman Group staff met on a separate occasion to review and revise the tool based on the faculty members’ experiences and expertise. The Freeman Group provided the tool at no cost in exchange for the residency program's evaluation of its reliability and validity.

The Freeman Group construct of nontechnical skills includes 4 domains identified by the acronym C.A.R.E.: *connect* with the patient, *adjust* the interaction to meet patient needs, *resolve* patient requests, and *empathize* with the patient. The Connect domain includes 3 assessments: attentiveness to patients, addressing patients appropriately, and customizing conversations to patients (eg, establishing rapport with patients or family members). The Adjust domain includes 2 assessments regarding the degree to which residents assess patient knowledge and use this assessment to influence their delivery of information and ensure patient understanding. The Resolve domain has 1 assessment that examines how well physicians detect and respond to patient requests. The Empathize domain also has 1 assessment related to the physician's ability to maintain a calm and respectful composure during demanding patient situations.

Each domain consists of subdomains that specifically label behaviors relevant to the domain. For example, the Connect domain that assesses physicians’ attentiveness has subdomains including physician never ignoring the patient, maintaining eye contact, and adjusting posture to be on the same level as the patient. The Connect domain that assesses customizing conversations to patients has the subdomains of attempting to establish rapport and addressing patient by last name and title at least once.

The Qualtrics dashboard allowed faculty to sign in on a smartphone or tablet, select the resident they are observing, and rate the resident during the interaction with a patient. Responses for each domain are acceptable, unacceptable, and not applicable. The tool uses logic, so if a resident is rated as unacceptable, a comment section opens to allow the faculty member to provide details. Unacceptable ratings also trigger a dropdown box listing the subdomains so that faculty can specify where the resident was deficient. Acceptable ratings on a domain do not warrant additional ratings on subdomains. However, comment boxes are provided at the end of each domain for faculty to provide feedback about acceptable interactions.

### Training Faculty and Examining Reliability

Freeman Group staff trained all faculty members of the emergency medicine residency program (n=12) on the constructs, use of the tool, and the Qualtrics platform. Training consisted of a 1-hour lecture followed by pre-filmed training videos during which faculty rated the simulation laboratory director's nontechnical skills in interacting with a simulated patient (eg, the simulation laboratory manager). The videos were recorded in the simulation laboratory using the simulation laboratory technology and were designed to present an array of competencies in nontechnical skills, ranging from excellent to average to poor performance. The faculty ratings were used to establish reliability measured as percent agreement.

We determined that reliability using a 3-tiered scoring system—superior, acceptable, and unacceptable—was not desirable, as faculty members demonstrated inconsistency in distinguishing superior behavior from acceptable behavior. Percent agreement on the domains ranged from 63% to 100%, with an average of 89.6%. Percent agreement on the subdomains ranged from 54% to 100%, with an average percent agreement for subdomains of 83.9%. After discussion with all faculty, we decided to convert the tool to a binary scoring system: acceptable or unacceptable. When a domain did not present during the interaction (eg, a patient did not make a request), faculty assigned the rating not applicable.

An additional 1-hour training session was scheduled to discuss the revised tool and measure reliability using the binary scoring system. Percent agreement among faculty members for 3 videos rated during training using the binary scoring system ranged from 56% to 100%, with an average of 93%. Percent agreement among faculty for the subdomains ranged from 55% to 100%, with an average of 97%.

### Rating Residents

Faculty rated residents during live observations of their patient interactions. Patient information was not recorded for formal analysis. Residents were aware that nontechnical skills were being observed and rated; however, as this evaluation was a baseline assessment, they were not given specific information about the rating tool or the nature of the constructs. Faculty observers were not working clinically in the ED during ratings. All ratings were completed during day shifts. The rating period was October 21, 2020, through January 25, 2021. Faculty rated the residents who were working in the ED when faculty were available. Ratings were tracked, and faculty attempted to adjust their schedules to ensure that all residents were observed at least once, but observations ranged from 1 to 6 per resident, with a mean of 3 observations per resident.

### Statistical Analysis

The percentages of responses for each domain were calculated. In the Results section, the percentages of responses are based on the available observations. For example, in the Resolve domain, 26 encounters did not involve a request and were rated as not applicable. Therefore, the percentage reported in the text is the percentage of acceptable ratings (78) divided by the available responses (84) rather than all 110 responses.

## RESULTS

Faculty completed 110 ratings of 110 unique patient encounters for 34 of 36 emergency medicine residents. Two residents did not work in the ED during the months the observations occurred. Percentages of acceptable ratings (excluding not-applicable responses) are presented in Table 1.

**Table 1.** Rating Percentages by Domain

Domain	Acceptable, n (%)	Unacceptable, n (%)	Not Applicable, n (%)
Connect			
Attentiveness	105 (95.5)	5 (4.5)	0 (0.0)
Speaking appropriately	97 (89.0)	12 (11.0)	1 (0.9)
Customized conversations	73 (68.9)	33 (31.1)	4 (3.6)
Adjust			
Delivery of information	94 (87.9)	13 (12.1)	3 (2.7)
Ensures understanding	100 (94.3)	6 (5.7)	4 (3.6)
Resolve			
Patient requests owned with urgency	78 (92.9)	6 (7.1)	26 (23.6)
Empathize			
Demonstrates respect and empathy	108 (98.2)	2 (1.8)	0 (0.0)

Note: The not-applicable data were not used to calculate the percentages shown for acceptable and unacceptable ratings. Those percentages were calculated by dividing the number of acceptable (or unacceptable) ratings by the sum of acceptable and unacceptable ratings. The percentages shown in the not-applicable column are based on the denominator of 110 (total number of ratings).

For the Connect domain, residents demonstrated appropriate levels of attentiveness during 95.5% of their patient interactions. They spoke appropriately to patients during 89.0% of the interactions. Most notably, residents’ ability to customize conversations to each patient and establish rapport was rated as acceptable in only 68.9% of patient interactions.

For the Adjust domain, residents delivered information at the patient's level 87.9% of the time, and residents ensured the patient's understanding 94.3% of the time. They resolved patient requests with urgency during 92.9% of the interactions. Residents were rated as demonstrating acceptable levels of empathy 98.2% of the time. Examples of acceptable and unacceptable behaviors that faculty noted in the comment boxes during observations are presented in Table 2.

**Table 2.** Comments on Unacceptable and Acceptable Behaviors by Domain

Domain	Unacceptable Behavior	Acceptable Behavior
Connect	TV was on and volume was up. This was distracting.	Did well to accommodate the patient's hearing difficulty. Got down to her level.
	Calls pt “bud.”	Dr XXX got close to patient, leaning down to pt level. Recognized the pt was uncomfortable and adjusted bed.
	Called the patient darling, dear, and sweetie.	
	There was a family member in the room who was never addressed.	
Adjust	Medical jargon used for medications and explanation of procedure.	Well explained after the mother asked question when she didn’t understand.
	Never asked if patient had questions or understanding.	Used “butt” and “belly” to improve patient understanding.
	Did not explain next steps or confirm understanding.	Asked if any questions.
Resolve	Did not explain why NGT was in place and what was needed before it could be removed when patient asked.	This was a patient transfer for admission that likely didn’t need to be admitted. The resident did a good job of not making the patient feel bad about being there.
	Pt requesting sedative. Resident does not address.	Pt was upset about not getting pain meds and the resident took ownership and followed up with nurse.
Empathize		Held hand during IV start.
		Excellent–wiped wound dry.

IV, intravenous [line]; NGT, nasogastric tube; pt, patient; TV, television.

## DISCUSSION

The purpose of the current study was to examine a novel assessment measure of nontechnical skills in emergency medicine residents. The C.A.R.E. domains developed in collaboration with the Freeman Group are consistent with studies that suggest patients seek empathy, attentiveness, and satisfactory communication with their physicians in the ED.^8,9^ In the current study, residents were typically rated as performing well on most C.A.R.E. domains. The tool demonstrated excellent agreement among faculty. However, anecdotally, faculty reported reluctance to rate interactions as unacceptable unless residents performed very poorly. Faculty frequently offered feedback for improvement when residents made minor mistakes, but they still rated the overall domain as acceptable. Our future research will examine alternative ratings that may better elucidate resident behavior and allow more constructive and accurate ratings of residents.

Our study demonstrates that the residents were able to empathize, resolve requests, demonstrate attentiveness, and ensure patient understanding >90% of the time. However, residents customized conversations to patients (eg, addressed patients appropriately and established rapport) <70% of the time. Establishing rapport and maintaining effective communication can be more difficult for emergency physicians than other medical specialists because of a number of environmental factors.^15^ Establishing rapport is particularly important as patient-centered care is emphasized.^10^ The Adjust domain had the second most unacceptable rating in terms of residents’ delivery of information at the patient's level (87.9%). These 2 findings are salient to our previous research demonstrating that physicians often fail to provide patients with pertinent information about their ED stay, procedures, or diagnoses.^16^ These findings suggest that education about maintaining rapport while assessing patient knowledge through verbal and nonverbal cues and learning to provide salient information that patients understand is necessary. Lack of understanding of their diagnoses, medications, return to the ED instructions, or follow-up plans can be dangerous for patients.^17^

Limitations of the current study include generalizability. This pilot study examined residents from 1 residency program at a single institution. Future research will expand the study population to other residency programs, other cities, and physicians at varying levels of training. In addition, some of the comment items require further investigation of patients’ perceptions. For example, whether calling patients names such as “bud” and “sweetie” is unacceptable may depend on patient variables, as well as geography. Further, kneeling down to the patient's level may make some individuals uncomfortable. Further work will involve examining patient reactions and interpretations of such behaviors. As mentioned previously, another limitation is the dichotomized ratings of acceptable and unacceptable. We are examining alternative scoring methods that better reflect resident performance. Finally, having faculty rate residents during interactions likely influences resident behavior, causing them to be mindful of their nontechnical skills. Despite these limitations, our data demonstrate areas for improvement in nontechnical skills.

## CONCLUSION

The current study found that during patient interactions, residents generally demonstrated empathy, resolved requests, demonstrated attentiveness, and ensured the patient's understanding; however, they demonstrated poorer performance in establishing and maintaining rapport and delivering information at the patient's level of understanding. Even when residents were mindful of faculty observing their nontechnical skills, they demonstrated a notable deficiency in this area. CMS mandates and ACGME milestones highlight the importance of positive patient-physician interactions, and the ability to establish and maintain rapport is essential. Appropriate delivery of information has been identified as a shortcoming in previous work by the authors. While nontechnical skills are an important area of focus in the literature, most of the work fails to examine nontechnical skills during interactions with patients. Our research suggests that it is not only imperative to attempt to measure nontechnical skills, but it is also essential to develop empirically supported interventions to improve skills in this area. This study provides important insight into nontechnical skill areas that may be influenced with intervention to improve patient interactions, and ultimately, influence patient satisfaction.

## Notes

*This article meets the Accreditation Council for Graduate Medical Education and the American Board of Medical Specialties Maintenance of Certification competencies for Patient Care, Medical Knowledge, Interpersonal and Communication Skills, and Practice-Based Learning and Improvement.*
